# Assessing the Reliability of Quantitative Fatty Acid Signature Analysis and Compound-Specific Isotope Analysis-Based Mixing Models for Trophic Studies

**DOI:** 10.3390/biom11111590

**Published:** 2021-10-27

**Authors:** Igor Prokopkin, Olesia Makhutova, Elena Kravchuk, Nadezhda Sushchik, Olesia Anishchenko, Michail Gladyshev

**Affiliations:** 1 Institute of Biophysics, Krasnoyarsk Scientific Center, Siberian Branch, Russian Academy of Sciences, 660036 Krasnoyarsk, Russia; prokop@ibp.ru (I.P.); phytolena@gmail.com (E.K.); labehe@ibp.ru (N.S.); hydrakr@rambler.ru (O.A.); glad@ibp.ru (M.G.); 2 Siberian Federal University, 660041 Krasnoyarsk, Russia

**Keywords:** CSIA-based mixing model, IsoError, QFASA, Daphnia, fatty acids, food

## Abstract

The study of the trophic relationships of aquatic animals requires correct estimates of their diets. We compared the quantitative fatty acid signature analysis (QFASA) and the isotope-mixing model IsoError, based on the compound-specific isotope analysis of fatty acids (CSIA-FA), which are potentially effective models for quantitative diet estimations. In a 21-day experiment, *Daphnia* was fed a mixture of two food items, *Chlorella* and *Cryptomonas,* which were supplied in nearly equal proportions. The percentages and isotope values of the FAs of the algal species and *Daphnia* were measured. The IsoError based on CSIA-FA gave an estimation of algae consumption using only one FA, 18:3n-3. According to this model, the proportion of consumption of *Chlorella* decreased while the proportion of consumption of *Cryptomonas* increased during the experiment. The QFASA model was used for two FA subsets—the extended-dietary subset, which included sixteen FAs, and the dietary one, which included nine FAs. According to both subsets, the portion of consumed *Chlorella* decreased from Day 5 to 10 and then increased at Day 21. The comparison of the two model approaches showed that the QFASA model is a more reliable method to determine the contribution of different food sources to the diet of zooplankton than the CSIA-based mixing model.

## 1. Introduction

The study of the trophic relationships and the foraging behavior of animals requires correct estimates of their diets. In addition to direct observations of feeding behavior, which are especially problematic for aquatic animals, there are established methods of diet estimation, e.g., visual (microscopic) analysis of stomach contents and feces. However, these approaches have several shortcomings. For instance, the analysis of stomach content may reveal mainly the indigestible parts of prey without showing easily digested food sources [1,2]. Moreover, many ingested types of prey stay viable after gut passage [3,4,5,6]. Furthermore, stomach contents and feces represent only recently consumed food sources rather than longer-term diet [2,7]. These limitations of the traditional methods can be overcome using stable isotopes and biochemical molecules, such as fatty acids (FAs), as well as their combination, through compound specific isotope analysis (CSIA), which is used to study assimilated food consumed over a comparatively long time span [7,8,9,10,11,12,13,14,15].

Stable isotope analysis (SIA) involves the measurement of the isotopic compositions of consumer tissues and probable food sources followed by calculation of consumer diets using isotope mixing models [16]. This method was used to establish the important role of terrestrial food sources in the diet of the freshwater pearl mussel (*Margaritifera margaritifera*) [17]. Additionally, isotope mixing models have enabled estimation of the total contribution of invasive quagga mussel (*Dreissena rostriformis bugensis*) to the diet of the invasive fish round goby (*Neogobius melanostomus*) and the native fish ruffe (*Gymnocephalus cernuus*) [18].

In most studies, where two isotope values, namely, δ^13^C and δ^15^N, are used, the isotope mixing model provides the mathematical solution for only three or fewer food sources [2]. To overcome this limitation, in the last decade, special Bayesian-based approaches have been developed for cases with many (>3) food sources [19]. This type of approach revealed changes in the diet, which included four food sources, of the freshwater shrimp *Paratya australiensis* inhabiting ecosystems differing in the availabilities of autochthonous and allochthonous organic matter [20]. The Bayesian models use probability distributions of the compounds (isotopes) to estimate the contributions of multiple food sources to the diets of consumers. However, the accuracy of the outcomes of these models is limited by the number of compounds used; an increase in the number of compounds used appears to be the only way to achieve reasonably accurate estimates of the contributions of multiple food sources [21].

An increase in the number of food compounds, up to ~40, may be achieved by using FAs instead of stable isotopes [2,7,13]. For instance, quantitative fatty acid signature analysis (QFASA) was successfully used to study the diets of different seal species [7], spectacled and Steller’s eider [9], Atlantic salmon [11], New Zealand sea lions [22], polar bears [23], flatfish [24], and steelhead trout [25]. A modification of the isotope mixing model, MixSIR, referred to as the FASTAR model, has been developed, which calculates the contribution of food sources to predator diets based on their FA composition [2]. Recently, a new generation of the Bayesian mixing model, MixSIAR, which employs a set of parameterizations that unify the MixSIR error structure, has been used with FA data to quantify the dietary components of spectacled and Steller’s eider, Atlantic salmon, tufted puffin, harp and harbour seals [13], and various fish species and aquatic invertebrates [26].

The FA biomarker-based approach was shown to provide better results to infer consumer diets than did the common two-tracer SIA approach [2]. However, there were no attempts made to compare the FA-based mixing models with a combination of the FA and SIA methods, namely CSIA, for the evaluation of the diets of aquatic consumers. Therefore, the aim of our study was to compare the application of QFASA with CSIA-based mixing models for inferring the diet of *Daphnia* in a controlled feeding experiment.

## 2. Materials and Methods

### 2.1. Cultivation of Organisms

The methods of cultivation are described in a study by Gladyshev et al. [27]. Briefly, a stock culture of *Daphnia galeata* Sars (collection of the Institute of Biophysics SB RAS, Krasnoyarsk, Russia), was maintained at 20–26 °C and fed *Chlorella vulgaris* Beijer (collection of the Institute of Biophysics SB RAS, Krasnoyarsk, Russia). For certain conditions of the experiment, *D. galeata* was fed both *C. vulgaris* and *Cryptomonas* sp. (collection of the I.D. Papanin Institute for Biology of Inland Waters RAS, Borok, Russia). Batch cultures of *C. vulgaris* and *Cryptomonas* sp. were grown at 18–22 °C and an illumination of 6000 lux (16:8 h light:dark cycle).

### 2.2. Preparation of Food

As in our previous work [27], algae from the batch cultures were concentrated by centrifugation and separated from the medium. The feeding mixture was prepared from the concentrated algae by dilution to obtain a concentration ~1 mg L^−1^ of organic carbon [10,27].

### 2.3. Experiments

The experiment was conducted under dim light (16:8 h light:dark cycle) at 20 °C. Six 1-L jars placed into a ‘plankton wheel’ (diameter, 38 cm, 0.2 rpm) [28] were used. In each jar, 418 ± 3 individuals of *Daphnia* of various ages and sizes were placed to simulate a natural population. The average biomass in each jar was 33.0 ± 1.6 mg of wet mass. Each day, 50% of the medium in each jar was replaced by a new portion of the algal feeding suspension to obtain a final concentration in the jars of ~1 mg L^−1^ of organic carbon.

Three runs of the experiment were performed. The first two runs lasted 10 days, while the third, Run III, lasted 21 days. In each run, in 2 jars the food was *Chlorella*, in 2 jars the food was *Cryptomonas*, and in 2 jars the food was a mixture of *Chlorella* and *Cryptomonas*, approximately 1:1 by organic carbon. The *Daphnia* fed *Chlorella*, *Cryptomonas*, and the mixture of these, were designated as *Daphnia* (Chl), *Daphnia* (Cry), and *Daphnia* (Mix), respectively.

**Figure 1:**
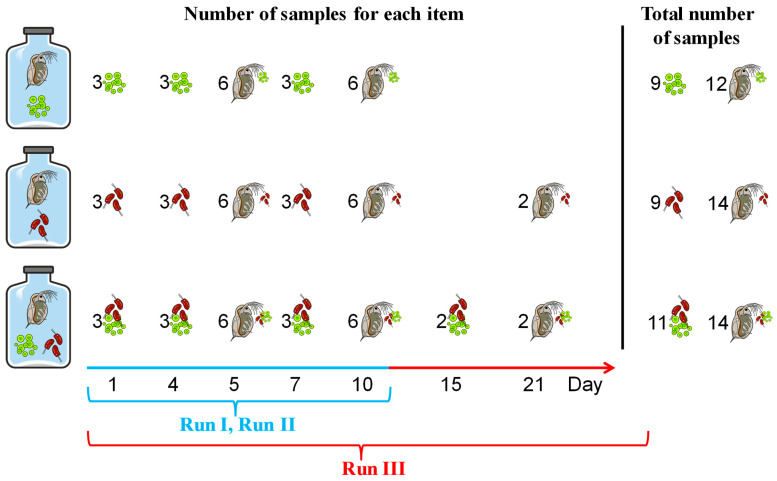
The experimental design with the number of samples taken for fatty acid and compound specific isotope analyses during the experiment.

Samples of algae for conducting the FA and CSIA analyses were taken from the batch cultures that were used for the preparation of food. Although the batch cultures were kept under the same stable conditions throughout the experiment and, therefore, were assumed to have similar FA and isotope compositions, samples (replicates) were collected from the batch cultures at different time points throughout the experiment, i.e., three samples were taken at Days 1, 4, and 7 of each run (Figure 1). In addition, two further samples were taken from the mixture at Day 15 in Run III (Figure 1). Thus, in total, 9 replicates were performed for each mono-species algae and 11 replicates for their mixture (Figure 1).

Samples of *Daphnia* for FA analysis and CSIA were taken from each 1-L ‘plankton wheel’ jar at Days 5 and 10 of each run (Figure 1). Additionally, two samples of *Daphnia* (Cry) and *Daphnia* (Mix) were taken at Day 21 in Run III (Figure 1). In total, 12 samples of *Daphnia* (Chl), 14 samples of *Daphnia* (Cry), and 14 samples of *Daphnia* (Mix) were taken (Figure 1). In addition, two samples from the stock culture of *Daphnia* were taken, designated below as *Daphnia* (0).

### 2.4. Fatty Acid Sampling and Analyses

The sampling of the algae cultures included their collection and conservation in chloroform:methanol (2:1, v:v), while the sampling of *Daphnia* included gutting, concentration, and conservation. The methods have been described in detail in our previous work [27].

The fatty acid analysis included homogenization of samples, lipid extraction, and fatty acid methylation. The methods have been described in detail elsewhere [29]. The description of the gas chromatograph and chromatographic and mass-spectrometric conditions can be found in studies by Gladyshev et al. [27,30].

### 2.5. Compound Specific Isotope Analyses

The analyses of compound specific isotopes were conducted using a Trace GC Ultra (Thermo Electron, Waltham, MA, USA) gas chromatograph coupled with a Delta V Plus isotope ratio mass spectrometer (Thermo Fisher Scientific Corporation, Waltham, MA, USA) via a type-III combustion interface. The method of CSIA-FA has been described in detail in studies by Gladyshev et al. [27,31].

### 2.6. Isotope Mixing Models

These models are based on the following system of equations:

(1) $$\sum_{i=1}^{n}f_{i}=1,$$

(2) $$\delta_{mix,j}=\sum_{i=1}^{n}f_{i}\cdot \left(\delta_{i,j}+\gamma_{i}\right),\;j=1\ldots m,$$

where *n* is the number of food sources, *m* is the number of isotopes, $f_{i}$ is the proportional contribution of the *i*-th food source to diet of the consumer, $\gamma_{i}$ is the isotope fractionation of the *i*-th food source, $\delta_{mix,j}$ is the *j*-th isotopic signature of the consumer, $\delta_{i,j}$ is the *j*-th isotopic signature of the *i*-th food source.

Equation (1) implies that there are no other food sources except the sources considered in it. Equation (2) shows the mass-balance composition for isotopic signatures of the consumer. For simplicity, $f_{i}$ is a constant for all *m* isotopes. Fractionation $\gamma_{i}$ is also assumed to be constant for all *m* isotopes [19].

When the number of food sources is less than or equal to the number of isotopes plus 1, the system of Equations (1) and (2) is solved with unique values of the proportional contributions of food sources to the diet of the consumer [32]. These contributions of food sources to the diet are quantified using different algebraic or geometrical methods [16,33].

In other cases, when the number of food sources exceeds the number of isotopes plus 1, the system of Equations (1) and (2) has no unique solution. There are several different approaches for estimating contributions of food sources to the diet in such situations. For example, the number of food sources can be reduced by combining them in groups containing the closest values of isotopic signatures or with some logical or biological connection or meaning [34]. Another approach is to use the mixing models with a Bayesian statistical framework, which enables estimation of the probability distributions of food sources in the diet of predators [19,35].

Most commonly, the isotopic compositions of the bodies or tissues of a consumer and food sources are used for model estimation of diet [16]. However, a simple two-source mixing model can also be applied for CSIA-FA to estimate the contributions of different food sources to the diet of laboratory-cultured *Daphnia* [10].

### 2.7. FA Selection for Isotope Mixing Modeling

Only FAs common both to food sources and to the consumer can be used for the isotope mixing model based on CSIA-FA. Furthermore, these fatty acids must be essential FAs, i.e., the consumer cannot synthesize them de novo. In our experiment, only three FAs met the above requirements: 16:3n-3, 18:2n-6, and 18:3n-3.

### 2.8. Isotope Mixing Model Routine

The first step in the model calculation was the correction of isotope values [36] (Table 2.1) of the food (algae) by adding fractionation coefficients to the measured average δ^13^C values of each FA. The fractionation coefficient for each individual FA *i* and for each individual diet *j*, Δδ^13^CFA*_ij_*, was calculated as the difference between the δ^13^C value of FA in *Daphnia* (δ^13^CFA*_Dij_*) and in its diet (δ^13^CFA*_ij_*) [10]:

Δδ^13^CFA*_ij_* = δ^13^CFA*_Dij_* − δ^13^CFA*_ij_* (3)

The second step was to calculate proportional contributions of the food sources to the diet of *Daphnia* using the mixing model IsoError by Phillips and Gregg [37]. The version for single isotope and two food sources was executed using Microsoft Excel (https://www.epa.gov/eco-research/stable-isotope-mixing-models-estimating-source-proportions, accessed on 17 April 2021).

### 2.9. Quantitative Fatty Acid Signature Analysis (QFASA)

QFASA is a statistical model that quantitatively estimates the predator diets using the FA composition of the predator and its food sources. The model takes a weighted mixture of the FA levels (% of total FAs) of the food sources and determines the mixture that most closely resembles the consumer FA composition [7]. The weighting coefficients that best explain the fatty acid composition of the predator correspond to the estimated proportion of the food sources in the predator diet.

Vector $\;\hat{y}$ (the theoretical proportions of ${\hat{y}}_{j}$ of each *j*-th fatty acid of the predator) is related to vector ${\hat{x}}_{k}$, consisting of the elements ${\hat{x}}_{kj}$ (the mean proportion of the *j*-th fatty acid of the *k*-th food source), via the following equation:

(4) $$\hat{y}=\underset{k}{\sum }p_{k}\cdot {\hat{x}}_{k}$$

where $p_{k}$ is the estimated proportion of the *k*-th food source in the predator diet.

The estimation task is to choose the $p_{k}$ values such that the model estimation $\hat{y}$ is as close as possible to the real measured profile y [7]. For this purpose, the Kulback–Liebler (*KL*) distance for comparing $\hat{y}$ and y is used:

(5) $$KL=\underset{j}{\sum }\left(y_{j}-{\hat{y}}_{j}\right)\cdot \log\left(\frac{y_{j}}{{\hat{y}}_{j}}\right)$$

The result of modelling is the set of $p_{k}$ that gives the smallest *KL* distance. This set is considered as the estimated diet of the predator.

### 2.10. Calculation of Calibration Coefficients for the QFASA Model

Following Iverson et al. [7], calibration coefficients, *CC*, are introduced to the model calculations to take into account consumer metabolism. These coefficients are determined for each common FA, and their values are obtained as the ratio of the amounts (levels) of the FAs in the consumers and in their food [11]:

(6) CC=FA level in consumer/FA level in food

In our experiment, two food items were used: *Chlorella* and *Cryptomonas*. Thus, two sets of *CC*s were calculated.

### 2.11. Selection of FA Subsets for the QFASA Model

A further concept, which is used in the QFASA model to increase the accuracy of calculation of diets, involves consideration of the fact that there are two groups of FAs: (1) “extended-dietary” FAs, i.e., the FAs synthesized by the consumer and obtained by consumption of food; (2) FAs of dietary origin [7]. Each FA selected for the model must be present at a level of >0.1% in at least one food item or in the consumer [11]. Based on the above conditions, in our experiment, two subsets of FAs were selected for the QFASA model calculations. The first subset included the “extended-dietary” FAs: 12:0, 14:0, 16:0, 16:1n-9, 16:1n-7, 16:2n-6, 16:2n-4, 16:3n-3, 16:4n-3, 18:0, 18:1n-9, 18:2n-6, 18:3n-3, 18:4n-3, 18:5n-3, 20:5n-3. The second subset included the dietary FAs: 16:2n-6, 16:2n-4, 16:3n-3, 16:4n-3, 18:2n-6, 18:3n-3, 18:4n-3, 18:5n-3, 20:5n-3.

### 2.12. The QFASA Model Routine

Four calculation scenarios were used: (1) *Chlorella CC* × 2 FA subsets; (2) *Cryptomonas CC* × 2 FA subsets. The model computations were performed in R 3.3.3 [38] (R Core Team, 2016), RStudio 1.0.136 [39] (RStudio Team, 2016), using the R package QFASA [7].

### 2.13. Statistical Analyses

Standard deviations (SD), standard errors (SE), Student’s *t*-test, and one-way ANOVA with Tukey HSD post hoc tests were calculated using STATISTICA software, version 9.0 (StatSoft, Inc., Tulsa, OK, USA).

## 3. Results

**Figure 2:**
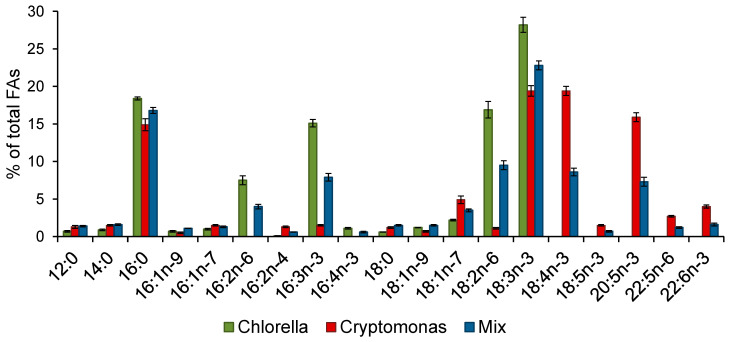
Mean values (± standard errors (SE)) of percentages of quantitatively prominent fatty acids (FAs) (% of total FAs) in biomass of *Chlorella vulgaris* (numbers of samples, *n* = 9), *Cryptomonas* sp. (*n* = 9) and their mixture (*n* = 11).

Across all samples, 43 FAs were identified. Quantitatively and qualitatively prominent FAs of the algae are shown in Figure 2. The percentages of all fatty acids in the biomass of each alga and their mixture were normally distributed. *Chlorella* and *Cryptomonas* had FA compositions typical for green and cryptophyte algae, respectively. Specifically, *Chlorella* had high levels of 16:2n-6, 16:3n-3, 18:2n-6, and 18:3n-3, while *Cryptomonas* had high levels of 18:4n-3, 20:5n-3, 22:5n-6, and 22:6n-3 (Figure 2). In the mixture, these FAs had intermediate levels (Figure 2), i.e., the mixture that was equalized by organic carbon (~1:1) appeared to be equalized also by the typical fatty acid composition (~1:1, Figure 2).

**Figure 3:**
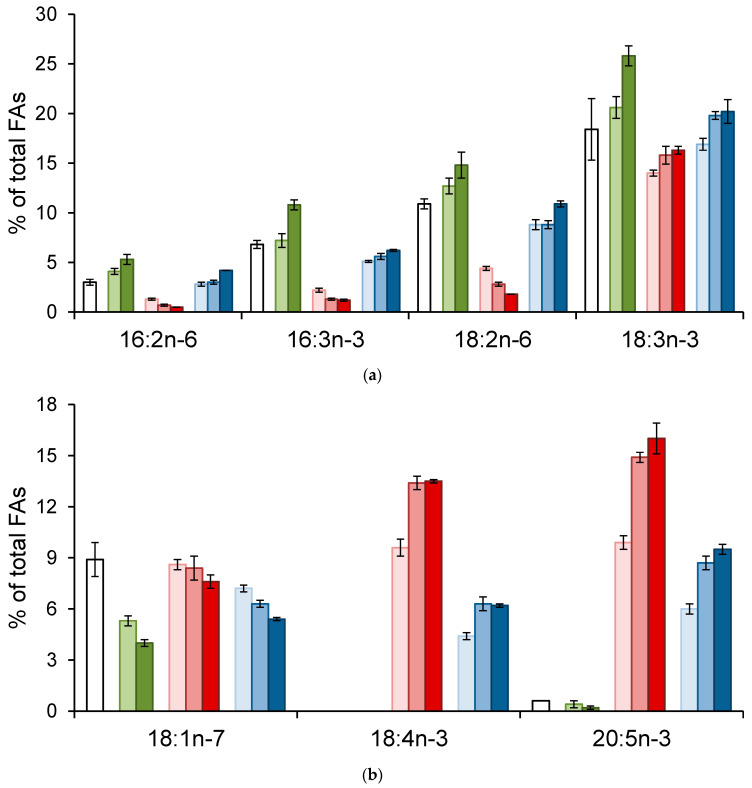

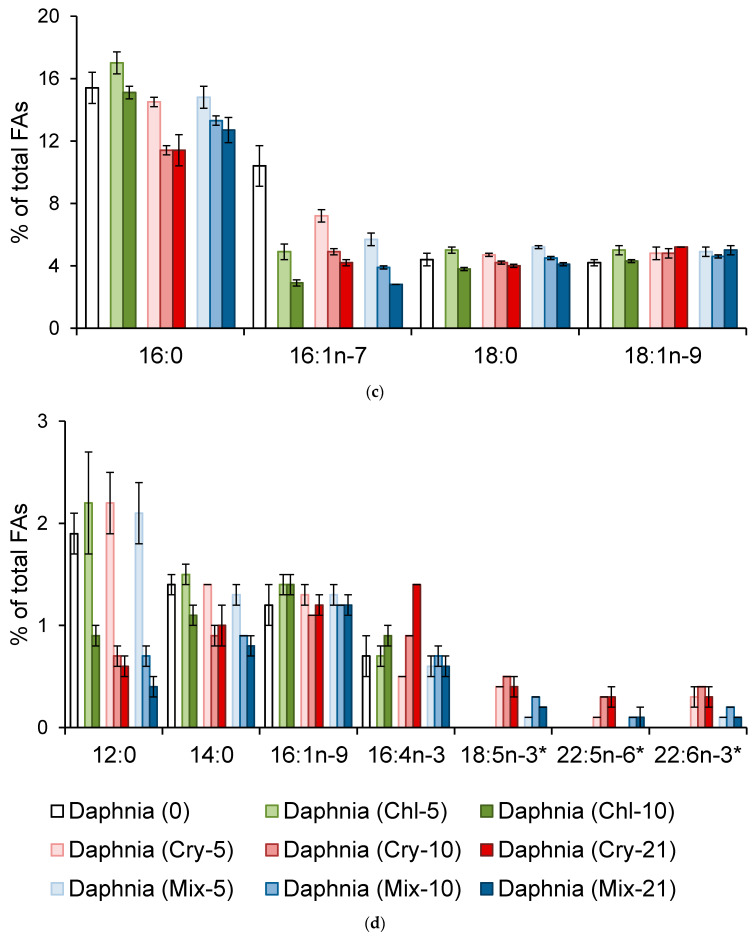
Mean values (% of the total FAs ± SE) of FAs in *Daphnia* fed *Chlorella* (Chl), *Cryptomonas* (Cry), and their mixture (Mix) at Days 5, 10, and 21 of the experiment, and in the stock culture (0). (**a**) FAs that were higher in *Chlorella*; (**b**) FAs that were higher in *Cryptomonas*; (**c**) dominant saturated and monounsaturated FAs; (**d**) minor FAs. Non-normally distributed variables are marked with *.

Quantitatively and qualitatively prominent FAs of *Daphnia* are given in Figure 3. Average percentages of 12:0, 14:0, 16:0, 16:1n-7, 18:0, and 18:1n-7 tended to decrease in all three treatments, *Daphnia* (Chl), *Daphnia* (Cry), and *Daphnia* (Mix), from Day 5 to 10 and to 21 (Figure 3b–d). By contrast, percentages of 18:3n-3 tended to increase during the course of the experiment in all treatments but the increase was only statistically significant in *Daphnia* (Chl) (*p* < 0.05) (Figure 3a). Percentages of 16:2n-6 and 16:3n-3 increased significantly at Day 10 in *Daphnia* (Chl) (*p* < 0.05) (Figure 3a). Stearidonic acid (18:4n-3) was not detected in *Chlorella* and *Daphnia* (Chl) but was abundant in *Cryptomonas* and increased significantly in *Daphnia* (Cry) and *Daphnia* (Mix) from Day 5 to 10 (*p* < 0.05) (Figure 3b). Similarly, 20:5n-3 was not detected in *Chlorella* and was present at a comparatively low level in *Daphnia* (Chl) but was abundant in *Cryptomonas* and increased significantly in *Daphnia* (Cry) and *Daphnia* (Mix) from Day 5 to 10 (*p* < 0.05) (Figure 3b). Percentages of 16:4n-3 increased significantly at Day 21 in *Daphnia* (Cry) (*p* < 0.05) (Figure 3d). The other FAs had no clear patterns of variations (Figure 3). The total FA content (mg/g, wet weight) in *Daphnia* was 6.9 ± 1.0 at the beginning of the experiments; in *Daphnia* (Chl)—8.1 ± 0.5 at Day 5 and 10.2 ± 0.7 at Day 10; in *Daphnia* (Cry)—9.8 ± 0.7 at Day 5, 10.6 ± 1.1 at Day 10, and 14.6 ± 1.5 at Day 21; in *Daphnia* (Mix)—8.1 ± 0.6 at Day 5, 9.8 ± 0.6 at Day 10, and 13.9 ± 0.4 at Day 21. Thus, the total FA content did not decrease in any treatment during the experiment, which implies that the *Daphnia* was not carbon-limited.

**Figure 4:**
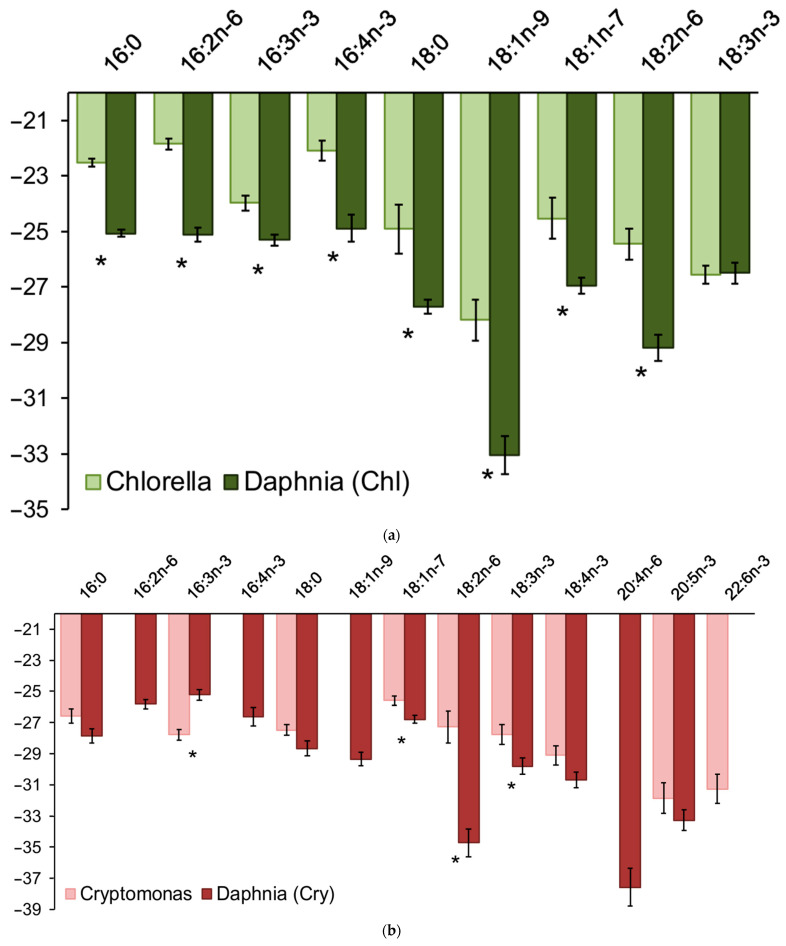

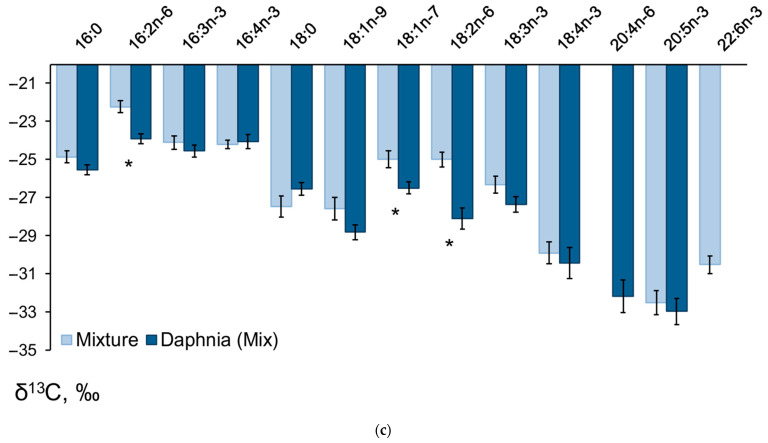
Average values of stable isotope values of FAs (± SE) in algae and in *Daphnia galeata*, fed (**a**) *Chlorella vulgaris*; (**b**) *Cryptomonas* sp.; (**c**) the mixture of algae. Significant differences according to *t*-test (*p* < 0.05) between algae and *Daphnia* are marked with *.

Values of the isotopes of all FAs in *Daphnia* were normally distributed. Statistically significant differences (fractionation) of the isotope values of many FAs were characteristic of *Daphnia* (Chl) (Figure 4a), *Daphnia* (Cry) (Figure 4b), and *Daphnia* (Mix) (Figure 4c).

Fractionation coefficients (Equation (3)) for the three FAs selected for the isotope mixing modelling are given in Table 1.

The stable isotope ratios of 16:3n-3 and 18:3n-3 gradually decreased during the experiment (Table 2). The stable isotope ratios of 18:3n-3 at Days 10 and 21 in Run III were depleted compared to Run I and Run II (Table 2).

Input data for the IsoError mixing model, including corrected isotope values of algae and isotope values of *Daphnia*, are provided in Table 3.

Results of the calculations of the proportions of the two algae consumed by *Daphnia* fed the 1:1 mixture are given in Table 4. Interpretable results were obtained only using 18:3n-3, while results based on 16:3n-3 and 18:2n-6 were non-interpretable (Table 4). According to the calculations, the proportion of consumption of *Chlorella* decreased, while the proportion of consumption of *Cryptomonas* increased from Day 5 to 21 of the experiment (Table 4).

Calibration coefficients (*CC*) calculated for the QFASA model are given in Table 5.

The QFASA model calculations for the subset of extended-dietary FAs resulted in the proportion of *Chlorella* in the diet of between 42.63%, when *CC* based on *Cryptomonas* were used, and 70.28%, when *CC* based on *Chlorella* were used (Table 6).

The model calculations for the subset of dietary FAs gave the proportion of *Chlorella* in the diet of between 43.43%, when *CC* based on *Cryptomonas* were used, and 70.95%, when *CC* based on *Chlorella* were used (Table 7). In all variants of calculations, the portion of consumed *Chlorella* decreased from Day 5 to 10 and then increased at Day 21 (Table 6 and Table 7).

The QFASA model calculations for the subset of extended-dietary FAs gave the proportion of *Cryptomonas* in the diet of between 29.72%, when *CC* based on *Chlorella* were used, and 57.37%, when *CC* based on *Cryptomonas* were used (Table 6). The model calculations for the subset of dietary FAs gave the proportion of *Cryptomonas* in the diet of between 29.05%, when *CC* based on *Chlorella* were used, and 56.57%, when *CC* based on *Cryptomonas* were used (Table 7). In all variants of the calculations, the proportion of consumed *Cryptomonas* increased from Day 5 to 10 and then decreased at Day 21 (Table 6 and Table 7).

## 4. Discussion

We conducted a simple feeding experiment under close to natural conditions by simulating the switching of *Daphnia* feeding from a single alga to two algae species in nearly equal proportions. In this simplest case, the tested models produced ambiguous results. The IsoError mixing model gave erroneous results when applied to isotope values of 16:3n-3 and 18:2n-6. These erroneous results could be explained in several ways. First, the signature difference between food sources should not be lower than 2‰ [37]. In our study, the isotope values of 16:3n-3 in *Chlorella* and *Cryptomonas* were almost equal. Therefore, we had abnormally high standard error values for this FA and an erroneous result. Second, according to Equation (2), the isotope values of consumers should be within the range of isotope values of their food sources. In our study, the isotope values of 16:3n-3 and 18:2n-6 of *Daphnia* were outside the range of the isotope values of these FAs in the algae. In such a situation, it is most often assumed that there is an additional food source that has not been considered or that there is uncertainty in the isotope values of the food and the consumer, which is caused by, e.g., sampling and measurement errors [16]. As a consequence, we had meaningless negative values for the isotope values of 16:3n-3 and 18:2n-6.

For 18:3n-3, the calculations might provide a more realistic picture, but three questions arise: (1) Why is there such a profound difference between results obtained using the essential 18:2n-6 and 18:3n-3 FAs? (2) Why did a ~3-fold decrease in the proportion of consumed *Chlorella* and a corresponding increase in the proportion of *Cryptomonas* occur? (3) Why were isotope values of 18:3n-3 in *Daphnia* so different in Run III and Runs I and II at Day 10 despite similar initial conditions of the runs?

We do not see any in principle differences between 18:2n-6 and 18:3n-3, since both these FAs cannot be synthesized by the consumer and, thus, must be obtained from food. Their roles in the consumer organisms are not clear, but they are likely to be the precursors of synthesis of long-chain FAs, 20:4n-6 and 20:5n-3, respectively, which, in turn, are the precursors of synthesis of eicosanoid signaling molecules [40,41,42,43,44]. Thus, if the IsoError, based on CSIA, were a reliable approach for the evaluation of diet, both 18:2n-6 and 18:3n-3 would give similar results.

With respect to the second question, the primary reason for the change in the proportions of consumed food items whose concentrations in the medium had been equal was selective feeding of the consumer. There is some evidence of selective feeding of *Daphnia*, including preferable consumption of *Cryptomonas* [4,45,46,47]. Nevertheless, if the model prediction of the ~3-fold decrease in the proportion of consumed *Chlorella* and the corresponding increase in the proportion of *Cryptomonas* is true, this change in food selection of *Daphnia* during the experiment is difficult to explain.

Concerning the third question, only Run III lasted until Day 21. Then, Run III significantly affected the final results of IsoError. However, in Run III, the isotope values of 18:3n-3 in Daphnia at Days 10 and 21 were the most depleted and closest to the isotope values of *Cryptomonas*. At present, we cannot explain the variability of isotope values between the runs that had similar initial conditions.

Because of the strong limitations in the selection of suitable fatty acids for the IsoError based on CSIA-FA, of the entire set of fatty acids, only one (18:3n-3) was found to be suitable. Since 20:5n-3, which is physiologically valuable for *Daphnia*, can be synthesized from 18:3n-3, its metabolic transformations could affect the isotopic ratios of 18:3n-3 in *Daphnia*. In the treatment where *Daphnia* consumed *Chlorella*, some of 18:3n-3 could probably be used for the synthesis of 20:5n-3, which is supported by the presence of 20:5n-3 in *Daphnia* (Chl) and the absence of this FA in *Chlorella*. In the treatment where *Daphnia* consumed *Cryptomonas*, the animals received a large amount of 20:5n-3 from food and did not need to synthesize this FA. The fractionation coefficients for 18:3n-3 obtained when growing *Daphnia* on a mono-species diet may be incorrect when used for a mixed-algal diet. The metabolic transformation of fatty acids is currently not well understood and deserves careful study [48]. Thus, differences in metabolic isotope fractionation of 18:3n-3 in *Daphnia* (Chl) and *Daphnia* (Cry) may cause misinterpretation of the results.

Since there are no reliable answers to the three above questions, or the erroneous results for the two FAs, the use of the IsoError model based on CSIA-FA seems to be unsuitable for evaluating the feeding spectra of zooplankton.

In the QFASA model for both subsets of FAs, namely extended-dietary FAs and dietary FAs, the proportion of *Chlorella* in the *Daphnia* diet decreased and then increased, while, in contrast, the proportion of *Cryptomonas* increased and then decreased. In the middle of the experiment (Day 10), the proportion of *Cryptomonas* consumed by *Daphnia* increased from ~30–45% to ~40–57%. This preference could be due to the requirement of *Daphnia* for 20:5n-3, since this FA is the determinant of growth and development for this genus [49,50,51,52,53,54,55]. When an optimal storage of 20:5n-3 was achieved, *Daphnia* increased the proportion of consumed *Chlorella* to ~48–65% (Day 21), since *Cryptomonas* is generally not a very good food for this genus [56,57]. The ranges presented cover the maximum variation in consumption of the two algae species, because calculations were performed with *CC*s based on *Chlorella* and *Cryptomonas*.

In contrast to IsoError, for QFASA, it was possible to use a large number of FAs, which is an important advantage of this method. For example, the two subsets of FAs for QFASA that we used were almost twice as different in the number of FAs but showed similar results. In addition, markers of both types of algae were used in QFASA, including those fatty acids that were found in only one of the species, thereby preventing underestimation of the proportion for one of the species.

Based on our findings, combined with data from the literature, we conclude that the QFASA model is the more reliable method to assess the contributions of different food sources to the diet of zooplankton.

## Figures and Tables

**Table 1:** Fractionation coefficients Δδ^13^CFA (‰) for the common essential dietary FAs selected for the isotope mixing model.

FA	Δδ^13^CFA	
	*Chlorella*	*Cryptomonas*
16:3n-3	−1.34	2.55
18:2n-6	−3.73	−7.44
18:3n-3	0.06	−2.03

**Table 2:** Stable isotope composition of FAs selected for isotope mixing modelling in *Daphnia galeata* fed the mixture of algae: average values (M, ‰) ± standard deviation (SD).

	16:3n-3	18:2n-6	18:3n-3
Day 5			
Run 1	−24.55	−30.37	−27.57
Run 1	−24.88	−31.73	−29.00
Run 2	−24.16	−28.76	−26.16
Run 2	−24.25	−28.83	−27.47
Run 3	−23.17	−25.07	−25.19
Run 3	−23.2	−26.26	−27.56
M ± SD	−24.04 ± 0.71	−28.50 ± 2.48	−27.16 ± 1.32
Day 10			
Run 1	−23.25	−29.78	−27.84
Run 1	−25.87	−27.88	−26.94
Run 2	−24.63	−27.51	−26.31
Run 2	−24.90	−26.48	−25.50
Run 3	−25.14	−27.00	−29.14
Run 3	−26.64	−27.55	−29.55
M ± SD	−25.07 ± 1.15	−27.70 ± 1.13	−27.55 ± 1.60
Day 21			
Run 3	−25.22	−29.31	−29.55
Run 3	−26.79	−27.79	−28.11
M ± SD	−26.00 ± 1.11	−28.55 ± 1.08	−28.83 ± 1.02

**Table 3:** Input data for IsoError mixing model: corrected average isotope values δ^13^Ccor (‰, ±SD) for algae and isotope values δ^13^C for *Daphnia*.

FA	δ^13^Ccor		δ^13^C		
	*Chlorella*	*Cryptomonas*	*Daphnia*		
			Day 5	Day 10	Day 21
16:3n-3	−25.31 ± 0.81	−25.23 ± 1.03	−24.04 ± 0.71	−25.07 ± 1.15	−26.00 ± 1.11
18:2n-6	−29.19 ± 1.66	−34.72 ± 3.10	−28.50 ± 2.48	−27.70 ± 1.13	−28.55 ± 1.08
18:3n-3	−26.50 ± 0.99	−29.80 ± 1.87	−27.16 ± 1.32	−27.55 ± 1.60	−28.83 ± 1.02

**Table 4:** Results of calculation by IsoError mixing model using isotope values of three common essential dietary FAs: average proportions of consumption of two algae species (M ± SE, %) by *Daphnia* at Days 5, 10, and 21 of the experiment.

FA	Alga	Source Proportion		
		Day 5	Day 10	Day 21
16:3n-3	*Chlorella*	−1491.1 ± 8523.1	−196.0 ± 1553.4	970.1 ± 5080.5
	*Cryptomonas*	1591.1 ± 8523.1	296.0 ± 1553.4	−870.1 ± 5080.5
18:2n-6	*Chlorella*	112.5 ± 21.7	127.0 ± 16.0	111.6 ± 17.9
	*Cryptomonas*	−12.5 ± 21.7	−27.0 ± 16.0	−11.6 ± 17.9
18:3n-3	*Chlorella*	80.1 ± 18.6	68.3 ± 21.7	29.4 ± 25.8
	*Cryptomonas*	19.9 ± 18.6	31.7 ± 21.7	70.6 ± 25.8

**Table 5:** Calibration coefficients (*CC*) for the QFASA model for *Chlorella* (Chl) and *Cryptomonas* (Cry) calculated from data presented in Figure 2 and Figure 3 (see Methods for details).

FA	CC (Chl)	CC (Cry)
12:0	1.26	0.46
14:0	1.24	0.66
16:0	0.82	0.77
16:1n-9	1.99	2.57
16:1n-7	2.77	2.80
16:2n-6	0.70	
16:2n-4	0.81	0.14
16:3n-3	0.72	0.76
16:4n-3	0.79	35.33
18:0	6.11	3.32
18:1n-9	3.55	7.82
18:1n-7	1.84	1.56
18:2n-6	0.88	1.66
18:3n-3	0.91	0.84
18:4n-3		0.70
18:5n-3		0.27
20:5n-3		1.01
22:5n-6		0.11
22:6n-3		0.08

**Table 6:** Estimation (M ± SD, %) of the diet of *Daphnia galeata* by the QFASA model using extended-dietary subset of FAs (12:0, 14:0, 16:0, 16:1n-9, 16:1n-7, 16:2n-6,16:2n-4, 16:3n-3, 16:4n-3, 18:0, 18:1n-9, 18:2n-6, 18:3n-3, 18:4n-3, 18:5n-3, 20:5n-3). *CC*—calibration coefficients for *Chlorella* (Chl) and *Cryptomonas* (Cry).

Days	CC (Chl)		CC (Cry)	
	*Chlorella*	*Cryptomonas*	*Chlorella*	*Cryptomonas*
5	70.28 ± 2.80	29.72 ± 2.80	54.25 ± 4.32	45.75 ± 4.32
10	60.99 ± 3.42	39.01 ± 3.42	42.63 ± 3.88	57.37 ± 3.88
21	65.21 ± 0.14	34.79 ± 0.14	48.02 ± 0.43	51.98 ± 0.43

**Table 7:** Estimation (M ± SD, %) of the diet of *Daphnia galeata* by the QFASA model using dietary subset of FAs (16:2n-6, 16:2n-4, 16:3n-3, 16:4n-3, 18:2n-6, 18:3n-3, 18:4n-3, 18:5n-3, 20:5n-3).

Days	CC (Chl)		CC (Cry)	
	*Chlorella*	*Cryptomonas*	*Chlorella*	*Cryptomonas*
5	70.95 ± 2.98	29.05 ± 2.98	56.91 ± 4.71	43.09 ± 4.71
10	60.76 ± 3.48	39.24 ± 3.48	43.43 ± 4.01	56.57 ± 4.01
21	64.72 ± 0.02	35.28 ± 0.02	48.32 ± 0.24	51.68 ± 0.24

## Data Availability

The datasets generated during and/or analyzed during the current study are available from the corresponding author on reasonable request.
